# Effectiveness of preoperative decolonization with nasal povidone iodine in Chinese patients undergoing elective orthopedic surgery: a prospective cross-sectional study

**DOI:** 10.1590/1414-431X20176736

**Published:** 2017-12-18

**Authors:** H-M. Peng, L-C. Wang, J-L. Zhai, X-S. Weng, B. Feng, W. Wang

**Affiliations:** Department of Orthopedic Surgery, Peking Union Medical College Hospital, Chinese Academy of Medical Sciences and Peking Union Medical College, Beijing, China

**Keywords:** Nasal methicillin-resistant, Staphylococcus aureus, Orthopedic surgery, Nasal povidone iodine, Prospective cross-sectional study

## Abstract

*Staphylococcus aureus* colonization in the nares of patients undergoing elective orthopedic surgery increases the potential risk of surgical site infections. Methicillin-resistant *S. aureus* (MRSA) has gained recognition as a pathogen that is no longer only just a hospital-acquired pathogen. Patients positive for MRSA are associated with higher rates of morbidity and mortality following infection. MRSA is commonly found in the nares, and methicillin-sensitive S. aureus (MSSA) is even more prevalent. Recently, studies have determined that screening for this pathogen prior to surgery and diminishing staphylococcal infections at the surgical site will dramatically reduce surgical site infections. A nasal mupirocin treatment is shown to significantly reduce the colonization of the pathogen. However, this treatment is expensive and is currently not available in China. Thus, in this study, we first sought to determine the prevalence of MSSA/MSRA in patients undergoing elective orthopedic surgery in northern China, and then, we treated the positive patients with a nasal povidone-iodine swab. Here, we demonstrate a successful reduction in the colonization of *S. aureus*. We propose that this treatment could serve as a cost-effective means of eradicating this pathogen in patients undergoing elective orthopedic surgery, which might reduce the rate of surgical site infections.

## Introduction

The rate of *Staphylococcus aureus* colonization in the nares of the general population is reported to be as high as 30% (1). In addition, genotyping studies reveal that as high as 80% of *S. aureus* infections are caused by the patient's own nasal flora (2). Furthermore, there is an association between the presence of nasal *S. aureus* and an increased risk of surgical site infection (SSI), which has been extensively demonstrated in orthopedic surgery (3–5).

Over the last 2 decades, methicillin-resistant *S. aureus* (MRSA) has gained recognition as a community-acquired pathogen and is no longer only a hospital-acquired pathogen. Importantly, MRSA is associated with higher rates of morbidity and mortality following infection (6).

The increased morbidity associated with SSI translates directly into increased costs associated with medical care. It is reported that surgical site infection is associated with a 2-week increase in hospital stay, double the rate of hospitalization, and triple the overall cost of treatment on average (3). Thus, screening and decolonization of *S. aureus* carriers prior to surgery has emerged as an important method for diminishing staphylococcal infections at the surgical site.

Surveillance studies (7–10) suggest that there is a high variation in the prevalence of MRSA infections depending on its location. Specifically, 0.6 to 6% of the general population has MRSA nasal colonization compared to methicillin-sensitive *S. aureus* (MSSA) nasal carriers, which make up 20 to 36.4% of the population. A previous review examining patients undergoing elective orthopedic surgery (11) concluded that screening initiatives needed to be intensified at all levels in order to contain the spread of this pathogen. Furthermore, it was emphasized that the screening initiatives at the regional and hospital levels would be the most effective.

An intranasal application of mupirocin is proven to be effective for the decolonization of this microbe and the prevention of invasive *S. aureus* infections in patients receiving long-term dialysis treatment (11–13). However, mupirocin is not currently available in China. In addition, due to its cost and the concern about patient compliance and the development of resistance, recent reports suggest that a preoperative nasal application of a povidone-iodine solution may be more efficacious than nasal mupirocin in preventing SSIs (14). The efficiency of the application of a nasal povidone-iodine swab for the decolonization of *S. aureus* is yet unknown in China.

Moreover, to the best of our knowledge, data regarding the prevalence and distribution of MSSA and MRSA in patients undergoing elective orthopedic surgery in China are lacking, and Chinese hospitals have no established guidelines for screening and decolonizing patients for the presence of MSSA and/or MRSA pathogens. Therefore, the main aim of this study was to assess the prevalence of MSSA/MRSA in the patients admitted to our institute. Furthermore, our secondary aim was to determine whether the current treatment protocols result in the successful decolonization of MSSA/MRSA.

## Patients and Methods

### Patients

This was a prospective cross-sectional study conducted in the Department of Orthopedics at Peking Union Medical College Hospital between August 2015 and February 2016. The eligible procedures included joint arthroplasty and spine fusion procedures requiring at least 3 days of overnight in the hospital. A consecutive series of patients undergoing elective orthopedic surgery during the above-mentioned interval participated in the present study. This study was approved by the Institutional Ethics Committee of Peking Union Medical College Hospital, and informed consent was obtained from the patients before the swabbing.

The minimum sample size needed was calculated to be 457 patients in order to detect the prevalence with a 2% precision, and this was based on the notion that the MRSA prevalence rate was expected to be 5%, as indicated in previous studies (11). The study subjects were patients who were admitted to the orthopedics ward within 24 h of admission to the hospital.

In addition, a standardized questionnaire was completed for each patient by a personal interview in order to evaluate the patients' characteristics and, therefore, assess possible risk factors and possible sources of transmission. The patient characteristics included gender, from urban or rural area, date of admission, reason for hospitalization, chronic underlying disease, presence of pets at home, smoking history, number of previous hospitalizations as well as the number of hospitalizations in the 3 months prior to this admission, antibiotic use in the 3 months prior to this admission (including the dosage, name and duration of use, if known) and hospitalization of a family member in the preceding 3 months (Table 1).

**Table 1.** Baseline characteristics of the screened patients.

Characteristic	Screened patients (n=545)
Mean age (years)	41.8±13.7
Male (%)	280 (51%)
ASA score at admission	
1-2	473/545
3	72/545
Underlying disorder	
Hypertension	134/545
History of smoking	142/545
Alcohol	70/545
Diabetes mellitus type 1 or 2	112/545
Renal insufficiency	22/545
Liver-function disorder	34/545
Malignant condition	12/545
Skin disease	31/545
Procedure	
Joint arthroplasty	307/545
Spine fusion	230/545
Other	8/545

ASA: American Society of Anesthesiologists.

### Exclusion criteria

Patients with any of the following were excluded from the study: hospitalization within past 3 months; a history of antibiotic use within the past 3 months; being in close proximity to other ill patients in the hospital or in their household; allergy to povidone iodine and an infectious indication for surgery.

### Screening

The patients were screened for nasal MRSA/MSSA colonization within 24 h of admission. The specimens were collected from both of the anterior nares by one cotton swab that was moistened with sterile saline. The walls of the vestibules of the anterior nares were thoroughly swabbed for 10–15 s. The swabs were then inoculated on both chromID MRSA agar (MRSA; BioMérieux S.A, France) and blood agar. The colonies suspected as MRSA were confirmed by MALDI-TOF MS (BioMérieux), and methicillin susceptibility was determined using cefoxitin-disk test. All of the patients in the study were then re-swabbed on the day of surgery using the same technique described above. This was done to determine whether the decolonization protocol (described below) eradicated MSSA/MRSA colonization.

### Decolonization

The patients who were positive for MSSA and/or MRSA underwent the decolonization procedure. For this, 5% povidone-iodine nasal swabs were used in both nostrils twice a day for 5 days prior to the surgery. The nurses prepped the patient's nostrils for approximately 30 s each using separate applicators. This process was then repeated using 2 additional applicators for a total application time of 1 min per naris (2 min total). The patients were also instructed to take chlorhexidine gluconate baths for the 5 days before the surgery. The patients who were negative for MRSA and MSSA colonization did not receive any decolonization treatment.

### Prophylaxis

The primary antimicrobial prophylaxis was cefuroxime (1.5 mg). The patients with a reported β-lactam allergy received clindamycin (600 mg), and those colonized with MRSA received vancomycin (1000 mg). Antibiotic infusion was started within 1 h of the incision (2 h for vancomycin) and was re-dosed according to the hospital guidelines. The patients were monitored for hospital-acquired *S. aureus* infection by means of microbiologic cultures. The physicians were encouraged to obtain culture samples if infection was suspected.

### Statistical analysis

All the statistical analyses were performed using the software SPSS (version 19.0; SPSS, Inc., USA). The statistical analyses comparing the pre-operative results to the results from the day of surgery were performed using the McNemar test. Statistical significance was defined as P<0.05.

## Results

### Patient screening

**Figure 1.**
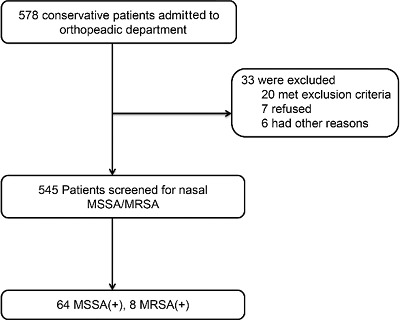
Study enrollment. MRSA: methicillin-resistant *S. aureus*; MSSA: methicillin-sensitive *S. aureus*.

During the study period, 545 patients underwent screening for both MRSA and MSSA by routine cultures. A total of 578 elective surgical procedures were performed during the same period, yielding a successful screening rate of 94.2%. Among the screened patients, 64 patients (11.74%) were identified as MSSA carriers, and 8 patients (1.28%) were identified as MRSA carriers. Thus, a total of 72 patients were treated with 5% povidone-iodine nasal swabs for decolonization (Figure 1).

### Decolonization results

The decolonization treatment was given for 5 days before the surgery. On the day of surgery, all patients (including the patients that were previously negative for MSSA and MRSA) were screened again to determine whether the MSSA or MSRA was decolonized from the patients' nostrils. The result from the day of the surgery screening showed that none of the patients were positive for MRSA colonization, showing a 100% successful decolonization. However, 11 patients were positive for MSSA colonization, which was 2% of the total patients (545) and was approximately a reduction of 92.6% compared to the screening before the decolonization, which was 11.74% of the total patients. Among the 11 patients that were positive for MSSA at the day of surgery screening, 7 were negative for MSSA before the decolonization treatment, and 4 had persistently positive MSSA colonization cultures. It should be noted that one of the patients who was persistently colonized for MSSA refused the decolonization protocol. Thus, there were only 3 patients who underwent the decolonization treatment and were persistently positive for MSSA at the time of surgery compared to 64 that were positive before the treatment. The eradication of the MSSA colonization was 94%, while the eradication of the MRSA colonization was 100% (Table 2). The compliance rate for the patients undergoing the decolonization protocol was 98.4% (only 1 patient refused).

**Table 2.** Comparison of screening results before and after decolonization.

	Pre-decolonization (n=545)	Post-decolonization (n=545)	P
MSSA cases	64 (11.7%)	3 (0.6%)	<0.001
MRSA cases	8 (1.3%)	0 (0)	<0.000

MRSA: methicillin-resistant *S. aureus*; MSSA: methicillin-sensitive *S. aureus*.

### Cost analysis

The average cost per patient for the MRSA screening was US$11 (SD, 0), and the average cost per patient for the hand-made povidone-iodine nasal swabs was US$1 (SD, 0; Table 3).

**Table 3.** Comparison of the cost (in US dollars) of different procedures.

	Screening	Decolonization
Culture+ mupirocin	$27.12	$130
PCR+ mupirocin	$121.16	$130
Our procedures	$10.00	$1

## Discussion

In this study, we assessed the prevalence of MSSA/MRSA in patients undergoing elective orthopedic surgery and treated the positive patients with 5% povidone-iodine nasal swabs to decolonize these pathogens from the anterior nares. The patients were screened within 24 h of their admission to the orthopedic department at our institute in northern China to determine the success of the decolonization. We chose to swab the anterior nares as they are the natural niches of *S. aureus* and are the most consistent areas from which the organism has been isolated (15). Other sites, such as the throat, axilla, groin and/or rectum, are alternative sites for colonization. However, these sites were excluded due to the concern about patient participation.

Two methods commonly used for the identification of *S. aureus* include culturing and polymerase chain reaction (PCR). Culturing the bacteria on chromogenic agar is a less expensive test, while PCR is considered the ‘gold standard' for MRSA detection (15–17 [Bibr B18] [Bibr B19] [Bibr B20] [Bibr B21]). Our decision to use the culture method was based on the economic standpoint (less expensive for the cost of material and labor) and the desired speed to provide the test result (1–2 days).

In our study, the prevalence of MRSA colonization was 1.28%, and the overall rate of *S. aureus* in the nasal region was 13.2%. Both of these rates were lower than the nasal carriage rates reported in other studies from China or abroad (1,9,18–22). MRSA incidence in elective orthopedic surgery was also lower than reported incidence in other surgeries (23,24). Costantini et al. (23) estimated a prevalence of 4.2% in children undergoing heart surgery. Another study by Ramirez et al. (24) reported a high rate of 6.4% in patients who underwent major gastrointestinal operations. Some previous large, population-based surveillance studies showed that colonization varies between different groups (ethnicity, gender, and age), with higher rates in whites, in men (7,9) and elderly patients (20). Our patients did not have a history of a recent hospital admission or other risk factors associated with MRSA colonization. The nasal swabs were collected within 24 h of admission to exclude colonization of the nares resulting from hospitalization. The screening population was different from other studies. Thus, the preoperative orthopedic outpatient colonization rates with MSSA and MRSA in our study population seem to reflect the real rates of the general population in Northern China.

The results of our study also demonstrated that utilizing a simple and less expensive decolonization protocol significantly reduced the colonization of MSSA/MRSA in nasal carriers. Only 3 of the patients who were positive for MSSA pre-operatively and underwent decolonization were persistently positive on the day of surgery. One patient refused to receive the decolonization protocol, and thus, the positive result on the day of surgery was expected. Another finding from the present study was that 7 of the patients who were positive for MSSA on the day of surgery were not positive during the pre-operative screen. The reason for this may be due to the fact that the pre-operative swabs were not sensitive enough to detect *S. aureus* colonization, and this might be improved by utilizing the PCR method instead of routine cultures (11,22) or by swabbing from multiple sites (15).

The application of nasal mupirocin to decolonize *S. aureus* and prevent subsequent SSIs is proven effective in many control studies (11,13,20,25,26). Chen AF et al. found that the decolonization protocols using intranasal mupirocin and chlorhexidine washes are effective for reducing MRSA/MSSA colonization (13). Decolonization of *S. aureus* using intranasal mupirocin and chlorhexidine in patients undergoing elective orthopedic surgery could significantly increase the risk of postoperative SSI (11,25). Agarwala et al. (20) reported that mupirocin was an effective treatment in clearing MRSA from the nares in adult patients undergoing orthopedic surgery in urban India, thereby reducing the incidence of MRSA SSI. In a double-blind, randomized, placebo-controlled study, use of mupirocin nasal ointment for perioperative eradication of *S. aureus* nasal carriage was significantly more effective, and the rate of endogenous *S. aureus* infections was 5 times lower than in the placebo group (26). However, nasal mupirocin is currently not available in China, and its compliance may be problematic due to its side effects and additional cost (14,27). A previous study suggested that preoperative hand-made nasal povidone iodine with topical chlorhexidine demonstrated a similar effectiveness, compared to nasal mupirocin, to decolonize *S. aureus* (13). The application of mupirocin to decolonize the nares of patients prior to orthopedic surgery was demonstrated as a cost-effective intervention. Currently, the nasal mupirocin available for application to the nasal mucosa cost approximately US$130/course (14), while our hand-made nasal povidone iodine cost approximately US$1/application, given the equal decolonization efficacy. Our method provides more value, as it is more suitable for developing countries, for universal surveillance and for the decolonization of patients undergoing elective orthopedic surgery.

Our study has several limitations. First, there is potential underestimation of the *S. aureus* carrier rate because we only focused on the anterior nares and did not include other sites, such as the throat, axilla, groin, and/or rectum. However, previous studies documenting a linkage between colonization and infection have cited nasal colonization, rather than reservoirs from these other sites, as the risk factor for infection. Second, our study design was cross sectional, which makes predictions of changes in colonization with time impossible. Third, this study only evaluated the effectiveness of the treatment using hand-made nasal povidone iodine swabs and a chlorhexidine body wash, but did not evaluate other treatment modalities, including oral antibiotics. Finally, this study detected the presence of MRSA/MSSA by a culture swab, rather than by PCR, which could increase the sensitivity of detection.

In conclusion, this study, for the first time, identified the prevalence of MRSA/MSSA in patients undergoing elective orthopedic surgery in Northern China. After identifying the positive patients, they were treated with a hand-made nasal povidone iodine swab, which successfully eradicated the detection of MSRA and significantly reduced the colonization of MSSA. This treatment was as effective as nasal mupirocin. However, mupirocin is not available in China at this time and it is significantly more expensive. We propose that a hand-made nasal povidone iodine swab should be evaluated in larger cohorts of orthopedic surgery patients to determine its efficacy in eradicating MRSA/MSSA colonization, which could significantly reduce SSI.
